# Management of Inflammatory Phlegmon in Appendicitis: Comparing the Role of Emergency vs. Interval Appendectomy at a Single Institution

**DOI:** 10.7759/cureus.73801

**Published:** 2024-11-16

**Authors:** Amos Nepacina Liew, Aparajita Tomar, Ashray Rajagopalan, Raelene Yi Mei Tan, Michelle Zhiyun Chen, Yeng Kwang Tay

**Affiliations:** 1 Department of Colorectal Surgery, Monash Health, Dandenong, AUS; 2 Department of Colorectal Surgery, John Hunter Hospital, Newcastle, AUS

**Keywords:** acute appendicitis, appendiceal phlegmon, appendicectomy, emergency appendicectomy, interval appendicectomy

## Abstract

Background

Acute appendicitis remains a common surgical pathology, with the accepted standard of care being appendectomy. However, in cases of acute appendicitis complicated by an inflammatory phlegmon, a dilemma remains regarding the best management options. The aim of our study was to examine the outcomes for patients with an appendiceal phlegmon, comparing emergency appendectomy with those who had initial conservative management followed by subsequent interval appendectomy.

Methods

We performed a retrospective analysis of all patients diagnosed with acute appendicitis with inflammatory phlegmon (January 2012 to December 2022), looking into the outcomes of patients managed with emergency appendectomy versus conservative management and subsequent interval appendectomy.

Results

A total of 127 patients were included in our study: 85 (66.9%) underwent emergency appendectomy, and 42 (33.1%) underwent interval appendectomy. Patients who underwent emergency appendectomy had a significantly shorter duration of symptoms compared to those undergoing interval appendectomy (two vs. seven days). Furthermore, there was a higher likelihood of either a partial cecectomy or ileocolic/right hemicolectomy in those undergoing emergency appendectomy (p=0.021). However, there was no difference in 30-day morbidity or mortality.

Conclusion

This study highlights the challenges in managing appendiceal phlegmons. We propose that interval appendectomies should be considered for patients who present with extensive phlegmonous appendicitis and a prolonged duration of symptoms.

## Introduction

Acute appendicitis remains a common surgical pathology in patients who present to the hospital with acute abdominal pain, with a lifetime risk of 8.6% in males and 6.9% in females [1]. It is diagnosed through a combination of clinical, biochemical, and radiological modalities, including ultrasound or computed tomography. Studies have shown that uncomplicated appendicitis can be treated with antibiotics; however, 15% of these cases will recur and require an operation [2]. The current gold-standard management for acute uncomplicated appendicitis is still appendectomy [3].

In complicated appendicitis, there is no consensus on the management algorithm, with some advocating for immediate surgical management and others for conservative management with delayed surgical intervention [4].

Acute complicated appendicitis is defined by an intra-abdominal abscess or inflammatory phlegmon secondary to acute appendicitis. Inflammatory phlegmons arise when there is a surrounding inflammatory mass that encompasses the appendix, adjacent viscera, and greater omentum [5]. It has been reported that appendiceal phlegmon occurs in 2-10% of cases of acute appendicitis [6], typically at the site of appendiceal perforation. It is routinely diagnosed radiologically through computed tomography scans of the abdomen and pelvis (CTAP). Aside from abdominal pain, patients are often hemodynamically stable, which deters surgeons from performing urgent surgery. However, this leaves surgeons facing a dilemma as to the ideal approach, conservative management with antibiotics and interval appendectomy, or semi-emergent surgery [7].

Our study aims to examine the outcomes of patients who underwent either approach to provide guidance for surgeons managing such a challenging issue.

This article was previously presented as a poster at the 2024 Royal Australian College of Surgeons' 92nd Annual Scientific Conference on May 6, 2024.

## Materials and methods

A retrospective analysis was performed on patients in our unit from January 2012 to December 2022 using electronic health records. Data were extracted by two authors (AL and AT). This retrospective study was approved by the Monash Health ethics committee (Reference no: RES-22-0000-547Q-89551).

All patients above the age of 18 diagnosed radiologically with acute appendicitis and appendiceal phlegmons who underwent either an emergency appendectomy or elective interval appendectomy in this study period were included. All patients included with phlegmonous appendicitis were diagnosed radiologically with a CTAP. All patients received intravenous antibiotics during their initial admission; the duration was dependent on their symptoms and corresponding blood markers.

Patients who had uncomplicated appendicitis or had an appendiceal phlegmon but did not undergo an appendectomy were excluded. Patients with appendiceal or colonic malignancy were also excluded.

Data collected included patient demographics, surgical approach, duration of symptoms, American Society of Anesthesiologists (ASA) score, type of surgery, time of surgery, operative times, length of stay, post-operative complications, and 30-day mortality and morbidity. The decision to proceed with an emergency or interval appendectomy for acute appendicitis was at the discretion of the consulting surgeon.

Outcomes

The primary outcome measure was the rates of post-operative complications and readmission rates after emergency and elective interval appendectomies in patients with appendiceal phlegmons. Secondary outcomes included length of stay, 30-day morbidity, and mortality.

Statistical analysis

Data analysis was performed using IBM SPSS Statistics (version 25, IBM Corp, Armonk, NY). Data were categorized into two groups based on whether they had an emergency or elective interval appendectomy following the diagnosis of an appendiceal phlegmon.

Continuous non-parametric data are expressed as medians with inter-quartile ranges, and categorical data are expressed as numbers with percentages, while continuous parametric data are expressed as means with SDs. The Shapiro-Wilk test was used to test for normality. Nominal or ordinal outcomes were compared using the Chi-square test, and continuous variables by the Mann-Whitney U or Student's t-test as appropriate. A p-value less than or equal to 0.05 was considered statistically significant.

## Results

Patient selection and characteristics

A total of 127 patients were diagnosed with an appendiceal phlegmon and underwent an appendectomy (emergency or interval). Eighty-five (66.9%) underwent emergency surgery, while 42 (33.1%) underwent interval appendectomy (Table 1). The median time to an interval appendectomy was 129 days (IQR 97.5-185.5).

**Table 1:** Patient demographics and operative details ASA: American Society of Anesthesiologists Score.

-	Emergency (n = 85)	Elective (n = 42)	P-value
Age, Median (Range)	47 (34-57)	43 (31-67)	0.9
Gender, Female, n (%)	42 (49.4)	17 (40.5)	0.342
Days of symptomology, Median (Range)	2 (1-4)	7 (5-14)	<0.01
ASA, n (%)	-	-	0.845
1	32 (37.6)	14 (33.3)	-
2	36 (42.4)	18 (42.9)	-
3	17 (20)	10 (23.8)	-
Surgical Approach, n (%)	-	-	0.663
Laparoscopic	80 (94.1)	41 (97.6)	-
Open	0	0	-
Laparoscopic converted to open	5 (5.9)	1 (2.4)	-
Operation performed, n (%)	-	-	0.021
Appendicectomy	72 (84.7)	41 (97.6)	-
Partial Caecectomy	5 (5.9)	0	0.17
Ileocolic Resection/Right Hemicolectomy	8 (9.4)	1 (2.4)	0.27
Operating time, Mean (SD), minutes	85 (38)	66 (29)	0.006
Primary operator, n (%)	-	-	-
Consultant/Fellow	63 (74.1)	21 (50)	-
Registrar	22 (25.9)	21 (50)	-
Length of stay post-op, Median (Range)	3 (2-5)	1 (1-2)	<0.01

There was no difference in median age (47 vs 43, p = 0.9), or gender (females; 42 (49.4%) vs 17 (40.5%), p= 0.342), between the two groups. The median ASA across the cohort was two, with no difference between the groups. However, there was a significant difference in the median number of days of symptomatology prior to diagnosis: two days in those who underwent emergency surgery versus seven days in those who underwent interval appendectomy (p < 0.01).

Peri-operative characteristics

Our study revealed that there was no statistical difference in the surgical approaches (laparoscopic, open, laparoscopic converted to open) between emergency appendectomy and interval appendectomy (p = 0.663) (Table 1).

However, emergency appendectomy was statistically a longer operation compared to interval appendectomy (mean time: 85 min vs 66 min, p< 0.01). The emergency appendectomy group also had a higher likelihood of conversion to either a partial cecectomy or ileocolic resection/right hemicolectomy (p= 0.021) (Table 1).

Post-operatively, there was no statistical difference in surgical site infections (Table 2). However, patients undergoing emergency appendectomy had a higher risk of post-operative ileus and had a longer length of stay compared to those undergoing interval appendectomy (3 vs 1 day, p<0.01).

**Table 2:** Clavien-Dindo classification of post-operative complications in emergency appendectomy vs. interval appendectomy. TOE: Transesophageal Echocardiography.

-	Emergency (n = 85)	Elective (n = 42)	P-value
I, n(%)	6 (7.1)	1 (2.4)	-
Post-operative ileus	5 (5.9)	0	0.042
Hypotension	1 (1.2)	1(2.4)	0.619
II, n (%)	3 (3.6)	0	-
Surgical site infection (SSI)	2 (2.4)	0	0.203
Atrial fibrillation - TOE cardioversion	1 (1.2)	0	0.369
III, n (%)	0	1 (2.4)	-
3b Return to theatre (Stump abscess washout)	0	1	0.135
IV, n (%)	0	0	-
V, n (%)	0	0	-

There were no reported mortalities between the two groups.

## Discussion

There has been ongoing debate regarding the role of emergency or interval appendectomy in the management of complicated appendicitis, with the advantages and disadvantages of both modalities represented in Table 3 [7].

**Table 3:** Advantages and disadvantages of emergency appendectomy vs. interval appendectomy.

Emergency Appendicectomy		Interval Appendicectomy	
Advantages	Disadvantages	Advantages	Disadvantages
1. Immediate source control	1. Increase risk of peri-operative morbidity	1. Easier delineation of anatomy	1. Failure of initial management
2. Comparatively shorter length of stay in hospital	2. Increase operative difficulty and need for further colectomy	2. Lower risk of peri-operative morbidity	2. Higher incidence of recurrent appendicitis
3. Mitigate risks of recurrent appendicitis	3. Increase risk of surgical site infections	-	3. Possibility of missed diagnosis of malignancy

Traditionally, in the context of an appendiceal phlegmon, interval appendectomy would have been performed after initial conservative treatment with IV antibiotics, lasting between 4 and 10 days [8]. Initial conservative management is theorized to localize the inflammatory process, decreasing the risk of surgical complications [6]. Although reports have shown that only 20% of patients treated conservatively for acute appendicitis experience recurrence, an interval appendectomy should still be performed as up to 10% of these patients might have another pathology, including Crohn’s disease and malignancy [9]. Furthermore, patients who have recurrent appendicitis after initial conservative management tend to pose a higher risk of peri-operative morbidity [10]. There has been a recent paradigm shift in the last decade, focusing on emergency appendectomy as a safe and viable option for managing an appendiceal phlegmon, especially with increasing surgeon experience in laparoscopic modalities [7]. In experienced hands, this can be associated with a shorter length of stay, reduced need for readmissions, and fewer additional interventions [11].

The majority (n=85 (66.9%)) of our patients with an appendiceal phlegmon underwent emergency surgery at our institution. This group of patients also had a much shorter duration of symptoms compared to those who underwent interval appendectomy. Patients with a longer duration of symptoms were postulated to have worse inflammatory changes, increasing the peri-operative and surgical risks if the patient were to undergo emergency surgery [12]. Hence, most of our surgeons opted for an interval appendectomy when patients presented with a history of 7 or more days of symptoms.

Our study also demonstrated a longer operative time for those who underwent emergency appendectomy. This could be due to the extensive inflammatory process, and the time required to define normal anatomy intraoperatively [13]. Furthermore, copious amounts of washout may have been required in those with intraoperative contamination [14]. The presence of an inflammatory phlegmon which involves the colon or small bowel also greatly increases the difficulty of the surgery, increasing the likelihood of a colonic resection [15]. This is evident from our study showing that 9.4% and 5.9% of patients undergoing emergency surgery had a right hemicolectomy and partial cecectomy respectively, compared to 2.4% (right hemicolectomy) in the interval appendectomy group. Moreover, patients undergoing an ileocolic resection or right hemicolectomy would face additional risks of anastomotic leaks and post-operative ileus [16].

Despite the longer operative time and higher risk of colonic resections during emergency appendectomy, our study demonstrated no statistical difference in surgical site infection or 30-day mortality.

Emergency appendectomy was associated with a higher post-operative ileus rate (p=0.042) and a longer post-operative length of stay (p<0.01). All 5 patients who had post-operative ileus in this group underwent laparoscopic appendectomy. Post-operative ileus could be attributed to intra-abdominal sepsis requiring extensive washout, adhesiolysis, and bowel handling.

This study highlights the difficulty in the management of appendiceal phlegmons. Our study demonstrated that emergency appendectomy in patients with inflammatory phlegmons have higher rates of peri-operative complications. Careful consideration should be given to those with large appendiceal phlegmons which might prove difficult to operate on in an emergent setting. Hence, the consideration for an interval appendectomy remains a viable option for patients with longer durations of symptoms and extensive inflammatory changes on CT scans.

Limitations

Our study has several limitations. The retrospective nature of the study has its limitations, where we are reliant on the accuracy of notes from our medical records and patients’ recall of symptoms. Furthermore, the interpretation of imaging is highly reliant on the radiologists' and surgeons' definition of an appendiceal phlegmon. Many of these imaging reports do not comment on the actual measurements of the inflammatory phlegmon, which could be a deciding factor for delayed surgery. Our relatively small sample size also suggests that the results, while significant, should be interpreted with caution and may not be generalizable to the general population.

## Conclusions

Our study highlights the challenges in managing complicated appendicitis with inflammatory phlegmons. There are multiple factors that clinicians need to consider, including the duration of symptoms, hemodynamic stability of the patient, and patient factors such as comorbidities. However, we propose that interval appendectomies can be considered for patients who present with extensive phlegmonous appendicitis and a prolonged duration of symptoms. This approach has the potential to mitigate peri-operative complications, including more extensive resections, post-operative ileus, and extended hospital stays. Emergency appendectomy can still be considered for a selected group of patients with non-extensive phlegmonous appendicitis or those who are septic and clinically unwell.
